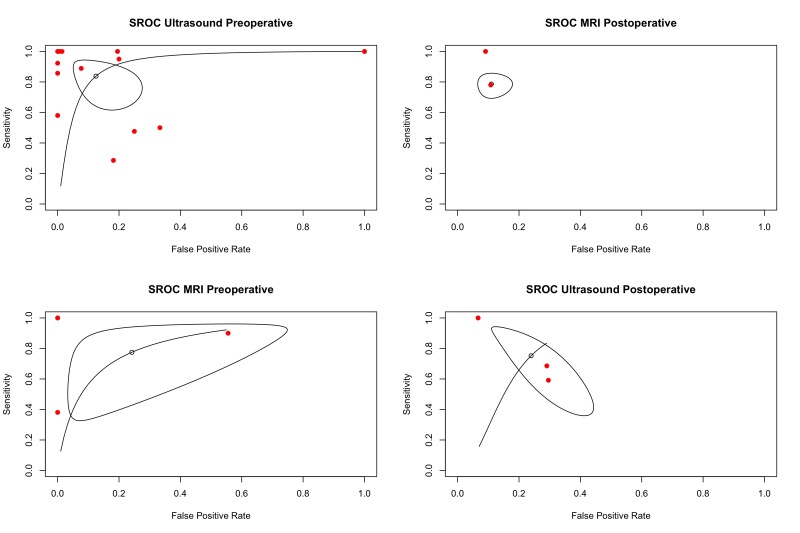# Accuracy of Ultrasound and MRI in Preoperative and Postoperative Management of Flexor Tendon Injuries: A Systematic Review and Meta-Analysis

**DOI:** 10.5435/JAAOSGlobal-D-25-00091

**Published:** 2025-11-03

**Authors:** David Sahai, Gilad Rotem, Ruby Grewal, Assaf Kadar

**Affiliations:** From the Schulich School of Medicine and Dentistry, University of Western Ontario, London, Ontario, Canada (Dr. Sahai, Dr. Rotem, Dr. Grewal, and Dr. Kadar); the Roth McFarlane Hand and Upper Limb Centre, London, Ontario, Canada (Dr. Rotem, Dr. Grewal, and Dr. Kadar); and the School of Medicine, Tel Aviv University, Tel Aviv, Israel (Dr. Rotem).

## Abstract

**Background:**

Complete and partial flexor tendon lacerations are challenging injuries to diagnose and manage. Imaging modalities can determine grade of laceration, and location of tendon ends preoperatively while detecting presence of adhesions, repair failure, and gap formation postoperatively. Despite these clear advantages, imaging modalities are underutilized because of issues with availability and concerns about accuracy.

**Methods:**

A systematic search of MEDLINE and Embase was conducted to identify papers examining the accuracy of ultrasonography (US) and MRI in preoperative and postoperative management of flexor tendon lacerations. COVIDENCE was used in blinded selection of papers for abstract and full-text review. R Studio was used for meta-analysis of pooled sensitivities and specificities, diagnostic odds ratios, and summary receiver operating curves of both US and MRI.

**Results:**

A total of 1197 papers were returned, with 40 being selected after full-text review and 24 being sufficient for statistical analysis. Significant heterogeneity existed for preoperative sensitivity of US and MRI, as well as preoperative specificity of US. MRI was more specific than US in the postoperative period (*P* < 0.01). Diagnostic odds ratios were >1 for all imaging modalities. The area under the curve for summary receiver operating curves in US preoperative, US postoperative, MRI preoperative, and MRI postoperative were 0.92, 0.81, 0.83, and 0.91, respectively.

**Conclusion:**

MRI is likely more specific than US in postoperative detection of tendon adhesions, tendon rupture, and gap formation following tendon repair. Notable heterogeneities exist in the literature, highlighting the future need for standardized comparisons of imaging modalities in preoperative management.

Clinical examination, imaging, and surgical exploration are all accepted methods of diagnosing flexor tendon lacerations on initial presentation.^1^ Still, previous literature demonstrates that even experienced hand surgeons and emergency physicians can miss as many as 30% of lacerations based on clinical examination alone.^2^ Furthermore, in properly identified injuries, the correct identification of width of tendon laceration does not improve with increased clinical experience.^3^

The variability in accuracy of physical examination alone underscores the need to explore imaging modalities in flexor tendon laceration identification including ultrasonography (US) and MRI. Preoperative imaging of flexor tendon lacerations provides opportunity to (1) determine laceration grade and avoid unnecessary surgery when tendon diameter is 50% preserved (Figure 1), (2) identify tendon stumps, and (3) avoid excessive surgical dissection to minimize operating room time.^4-7^ Postoperatively, imaging is an important adjunct in decision making when trying to discern tendon adhesions from repair failure.

**Figure 1:**
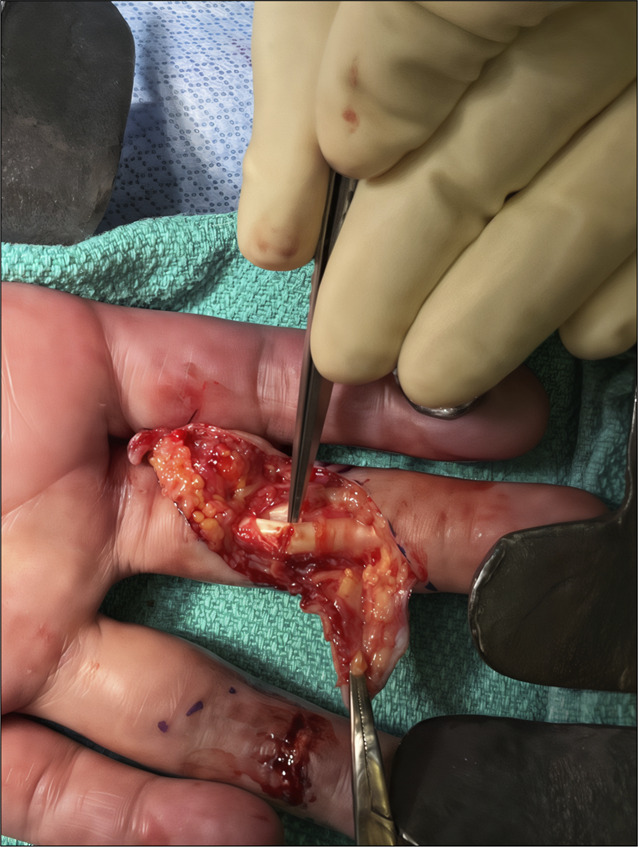
Image showing partial laceration of the flexor digitorum profundus of the middle finger. The patient presented clinically as a full flexor laceration and intraoperatively a partial tear of 40% was discovered.

Despite the proposed benefits of US and MRI in flexor tendon injury management, their accuracies in the literature are inconsistent. Preoperatively, diagnostic sensitivity of flexor tendon laceration has been cited as low as 50% and as high as 100% with US; similarly, MRI sensitivity has been cited anywhere between 38% and 100%.^1,8,9^ Postoperatively, US sensitivity in identification of flexor tendon repair integrity has been reported between 60% and 100%.^10,11^

Given the heterogeneity in reported accuracies of both US and MRI, we sought to conduct a systematic review and meta-analysis to obtain true values for sensitivity and specificity of both modalities in preoperative and postoperative management of partial and complete flexor tendon lacerations. We hypothesized that MRI would be markedly more accurate than US in preoperative and postoperative identification of flexor tendon injuries.

## Methods

### Registration

This project was registered on Open Science Framework on March 13^th^, 2024, and the research protocol can be located at the following link: https://osf.io/krxf4/?view_only=9f08dfca8506437caf0f1fa808b20e98.

### Search Strategy

A systematic search of MEDLINE and Embase from January 1, 1946, to March 8, 2024, was developed using search strategies developed by AI, who is a clinician librarian with extensive experience in electronic literature searches. AI developed these search strategies in cooperation with authors AK and DS using a combination of medical subject headings (MeSH) in MEDLINE and Emtree headings in Embase outlined in Appendix A, http://links.lww.com/JG9/A446. Twelve pertinent papers were identified between AK and DS to inform the search strategy developed by AI. A gray literature search of google scholar was also implemented to ensure no papers were missed that were not registered in these databases. The articles in the reference sections of included papers were also considered for inclusion.

Inclusion and exclusion criteria for articles were developed using the participant-concept-context framework.^12^ Our patient population included human and cadaveric patients 18 years or older with partial or complete flexor tendon laceration in any of the flexor tendon zones. Selected papers evaluated the efficacy and accuracy of imaging modalities for either preoperative or postoperative management of flexor tendon lacerations. Studies included patients in acute care or primary care settings and comprised prospective and retrospective research. The papers were written in English between 1946 and 2024. Exclusion criteria included patients <18 years of age; secondary research, including literature reviews, meta-analysis, and systematic reviews; papers not written in English; and papers where there was trauma to the flexor tendons without the presence of partial or complete laceration.

### Statistical Analysis

Statistical analyses were informed by the guidelines proposed by Pambabay-Calero et al^13^ for conducting a meta-analysis in diagnostic tests of low-prevalence diseases using a univariate analysis. Pooled sensitivities and specificities, as well as their 95% confidence intervals, were calculated using the “binom” package in *R* Studio. The “metaprop” function in the “meta” package was used to aggregate proportions between studies using a random-effects model. The “meta” package was also used for generation of forest plots for the sensitivities and specificities calculated via the “binom” package. Diagnostic odds ratios were developed from true-positive (TP), true-negative, false-positive (FP), and false-negative (FN) values using the “madauni” function in the “MADA” library. Using the “MADA” library, diagnostic odds ratios were calculated to provide a measure of discriminative diagnostic performance, with a value greater than 1 suggesting that the chosen imaging modality can more accurately distinguish injured from uninjured patients when compared with chance alone. The “MADA” library was then used for generation of spontaneous receiver operating curves (sROCs). sROCs provided a plot of sensitivity vs. (1—specificity) across the included studies to provide a visual representation of the trade-off between sensitivity and specificity. A perfect test places all values at [0, 1] with an area under the curve (AUC) of 100. A greater AUC increases the likelihood that a TP will be detected by the imaging modality in question. Typically, an AUC greater than 0.8 is generally accepted as good, and an AUC greater than 0.9 is deemed excellent.

Finally, the “metabin” function in the “meta” package was used to detect statistically significant differences between (1) imaging modality sensitivities and specificities both preoperatively and postoperatively and (2) partial and complete tendon laceration sensitivities and specificities in the preoperative period for US. For all statistical tests, statistical significance was reached at either (1) a *P* value of <0.05 or (2) 95% confidence intervals that did not overlap between imaging modalities.

## Results

### Article Selection

**Figure 2:**
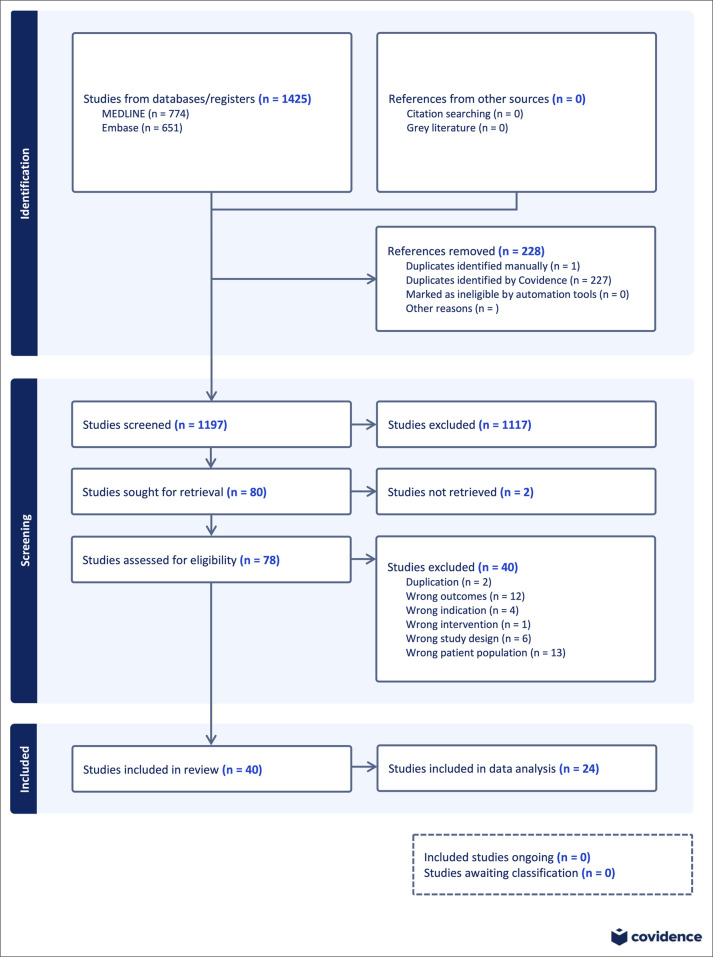
PRISMA flow diagram demonstrating phases of systematic review (modified from COVIDENCE PRISMA figure).

A flow diagram outlining the process of article identification, screening, and inclusion is provided in Figure 2. A total of 1197 papers were included in the abstract and text screening, with 78 receiving full text-review for eligibility. In the title and abstract screening phase, D.S. and G.R. screened papers independently with a proportionate agreement of 0.96 and a Cohen Kappa Coefficient of 0.70, indicating substantial agreement (1197 papers). D.S., A.K., and G.R. all participated in the full-text screening review of 78 articles with Cohen's Kappa Coefficients of 0.50 between A.K. and D.S. (50 decision combinations) and 0.84 between D.S. and G.R. (49 decision combinations). A.K. and G.R. both decided on one additional paper with a random agreement probability of 1.0. Forty papers were selected for inclusion, and 24 were included for meta-analysis. All 24 papers included imaging of the tendon (either US or MRI) followed by validation of the findings in the operating room or cadaveric dissection. Papers selected for inclusion in meta-analysis contained enough reported events (ie, TP, true negative, FP, FN) to calculate a sensitivity and specificity. Preoperatively, 15 papers assessed accuracy of US and four assessed accuracy of MRI. In the postoperative period, three papers assessed accuracy of US and two assessed accuracy of MRI. In the included studies, three (Bezirgan et al^1^; Stephens et al^9^; Renfree et al) used both US and MRI on the same subjects to allow direct comparison of diagnostic performance in identical clinical conditions. Table 1 provides outcomes from our included studies and Appendix B, http://links.lww.com/JG9/A447 provides their citations.

**Table 1:** Imaging Modalities, Type of Injury, Preoperative and Postoperative Outcomes, Sensitivities, and Specificities for Articles Selected From Full-Text Review

Authors	Population Type (Cadaveric vs. Human)	Imaging Modality	Type of Injury	True Positive	True Negative	False Positive	False Negative	Sensitivity	Specificity
Preoperative									
Bezirgan et al, 2023^2^	Human	MRI	Complete and partial laceration	10	1	0	0	1	1
Kumar et al, 2000^15^	Human	MRI	Complete laceration	4	0	0	0	1	NA
Rubin et al, 1996^16^	Human	MRI	Complete laceration	6	0	0	0	1	NA
			Intact	4	0	2	0	1	0
			Partial (<50%)	1	0	0	0	1	NA
			Partial (>50%)	2	0	3	0	1	0
Stephens et al, 2021^10^	Cadaveric	MRI	Partial laceration	8	11	0	13	0.38	1
		US	Partial laceration	6	9	2	15	0.29	0.82
Drapé et al, 1998^17^	Human	MRI	Rupture	12	NA	NA	NA	NA	NA
Nho et al, 2013^18^	Human	MRI	Rupture	2	NA	NA	NA	NA	NA
		US	Rupture	1	NA	NA	NA	NA	NA
Matloub et al, 1996^19^	Human	MRI	Rupture	6	4	0	0	1	1
Akhavan et al, 2019^9^	Human	US	Complete and partial laceration	48	165	1	0	1	0.99
Bekhet et al, 2021^20^	Human	US	Complete and partial laceration	30	69	1	0	1	0.99
Lee et al, 2000^5^	Human	US	Complete laceration	6	12	1	1	0.86	0.92
	Human	US	Partial laceration	2	NA	NA	NA	NA	NA
Bezirgan et al, 2023^2^	Human	US	Complete and partial laceration	6	2	1	6	0.50	0.67
Meisami 2019^21^	Human	US	Complete and partial laceration	73	26	10	4	0.95	0.72
Alvarez et al, 2019^22^	Cadaveric	US	Complete laceration	96	NA	NA	NA	NA	NA
Ravnic et al, 2011^23^	Cadaveric	US	Complete laceration	47	31	0	34	0.58	1
Zhang et al, 2012^24^	Human	US	Complete laceration	79	NA	0	NA	NA	NA
			Partial laceration	16	NA	0	NA	NA	NA
Baz et al, 2021^25^	Human	US	Complete laceration	10	NA	NA	NA	NA	NA
			Partial laceration	7	NA	NA	NA	NA	NA
Jeyapalan et al, 2008^26^	Human	US	Complete laceration	2	0	1	0	1	0
Lee et al, 2018^27^	Human	US	Complete laceration	11	NA	NA	NA	NA	NA
Al-Hourani et al, 2018^28^	Human	US	Complete laceration	12	7	0	1	0.92	1
Kazmers et al, 2017^29^	Cadaveric	US	Partial laceration	10	6	2	11	0.48	0.75
Kim et al, 2022^30^	Human	US	Partial laceration	12	66	16	0	1	0.80
Read et al, 1996^31^	Human	US	Rupture	6	4	0	1	0.86	1
Wang et al, 1999^32^	Human	US	Rupture	3	3	NA	NA	NA	NA
Sugun et al, 2010^33^	Human	US	Rupture	14	0	1	0	1	0
Gilleard et al, 2010^34^	Human	US	Rupture	57	4	1	3	0.95	0.80
Leonard et al, 2011^35^	Human	US	Rupture	13	NA	NA	NA	NA	NA
Sunagawa et al, 2003^36^	Human	CT	Complete and partial laceration	12	6	0	1	0.92	1
Authors	Population type (cadaveric vs. human)	Imaging Modality	Complete or partial laceration versus rupture	True positive	True negative	False positive	False negative	Sensitivity	Specificity
Post-operative									
Hinckley et al, 2023^11^	Cadaveric	US	Tendon gapping	42	50	21	29	0.59	0.70
Nugent et al, 2012^37^	Human	US	Tendon thickness and glide	NA	NA	NA	NA	NA	NA
Puippe et al, 2011^38^	Human	US	Shape, excursion, and scar tracking	NA	NA	NA	NA	NA	NA
Budovec et al, 2006^12^	Human	US	Rerupture and adhesions	13	14	1	0	1.00	0.93
Reissner et al, 2018^39^	Human	US	Tendon gapping, bowstringing, and repair integrity	NA	NA	NA	NA	NA	NA
Sun et al, 2022^40^	Human	US	Complete rupture	7	NA	NA	1	0.88	NA
Drape et al, 1996^41^	Human	MRI	Rerupture and adhesions	19	20	2	0	1.00	0.91
Renfree et al, 2021^42^	Cadaveric	1.5T MRI	Tendon gapping	54	75	8	15	0.78	0.90
		3.0T MRI	Tendon gapping	56	74	10	16	0.78	0.88
		US	Tendon gapping	48	61	25	22	0.69	0.71
Renfree et al, 2021^42^	Cadaveric	Static US	Partial	23	25	7	20	0.53	0.78
		Dynamic US	Partial tear	24	25	7	19	0.56	0.78

Refer Appendix B, http://links.lww.com/JG9/A447 provides their reference citations.

### Pooled Results

Pooled sensitivities and specificities with heterogeneity measures for both US and MRI both preoperatively and postoperatively are summarized in Table 2.

**Table 2:** Pooled Sensitivities and Specificities With Heterogeneity Measures for US and MRI Preoperatively and Postoperatively for Both Partial and Complete Tendon Injuries

	US Preoperative	MRI Preoperative	US Postoperative	MRI Postoperative
Sensitivity				
Value [CI]	0.85 [0.69-0.94]	0.82 [0.42-0.97]	0.65 [0.55-0.75]	0.89 [0.46-0.99]
I^2^	79%	76%	59%	64%
τ^2^	2.28	2.33	0.04	1.82
Heterogeneity *P* value	<0.01^a^	<0.01^a^	0.04^a^	0.12
Specificity				
Value [CI]	0.89 [0.77-0.95]	0.77 [0.39-0.95]	0.72 [0.64-0.81]	0.89 [0.84-0.93]
I^2^	59%	48%	29%	0%
τ^2^	1.47	1.43	0.00	0.00
Heterogeneity *P* value	<0.01^a^	0.12	0.25	0.81

CI = confidence interval

a Statistically significant.

**Figure 3:**
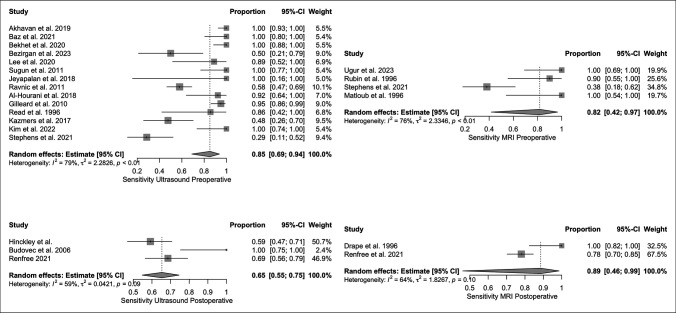
Sensitivities for US and MRI in diagnosis of flexor tendon injuries both preoperatively and postoperatively.

**Figure 4:**
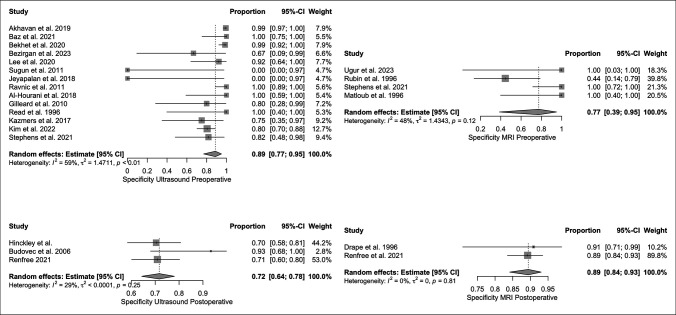
Specificities for US and MRI in diagnosis of flexor tendon injuries both preoperatively and postoperatively.

The pooled sensitivities of US and MRI for preoperative and postoperative identification of flexor tendon lacerations are outlined in Figure 3. Pooled preoperative and postoperative specificity values for imaging modalities are outlined in Figure 4. For the calculations of pooled sensitivity in both US and MRI preoperatively, heterogeneity was significant. Postoperative sensitivity calculations in US were significant but in MRI revealed insignificant heterogeneity. Regarding specificities, US preoperatively had significant heterogeneity, while US postoperative and MRI pre and postoperative did not.

Using a random-effects model, the pooled sensitivity of US in preoperative identification of flexor tendon injuries was 0.85 [0.69 to 0.94] with I^2^ and τ^2^ values of 79% and 2.28, respectively (*P* < 0.01). The pooled sensitivity for MRI preoperatively was 0.82 [0.42 to 0.97] with I^2^ and τ^2^ of 76% and 2.33, respectively (*P* < 0.01). The pooled sensitivities for US vs MRI preoperatively were not statistically significant (*P* = 0.81).

The postoperative sensitivity for US was 0.65 [0.55 to 0.75] with an I^2^ and τ^2^ of 59% and 0.04, respectively (*P* = 0.04). Postoperative MRI sensitivity was 0.89 [0.46 to 0.99] with an I^2^ and τ^2^ of 64% and 1.83, respectively (*P* = 0.12). There were no statistically significant differences between US and MRI specificity postoperatively (*P* = 0.39).

Regarding preoperative specificities, heterogeneity was significant in US but not in MRI. US in the preoperative period had a specificity of 0.89 [0.77 to 0.95] with an I^2^ and τ^2^ of 59% and 0.04% (*P* < 0.01). MRI in the preoperative period had a specificity of 0.77 [0.39 to 0.95] with an I^2^ and τ^2^ of 48% and 1.43, respectively (*P* = 0.12). There were no statistically significant differences in preoperative specificities of US as compared with MRI (*P* > 0.05).

Regarding postoperative specificities, neither US nor MRI had significant heterogeneities. Postoperative US specificity was 0.72 [0.64 to 0.81] with an I^2^ and τ^2^ of 29% and <0.01, respectively (*P* = 0.25). Postoperative MRI had a specificity of 0.89 [0.84 to 0.93] with an I^2^ and τ^2^ of 0% and 0.00, respectively (*P* = 0.81). MRI was significantly more specific than US in the postoperative period in identification of flexor tendon injuries (*P* < 0.01).

**Figure 5:**
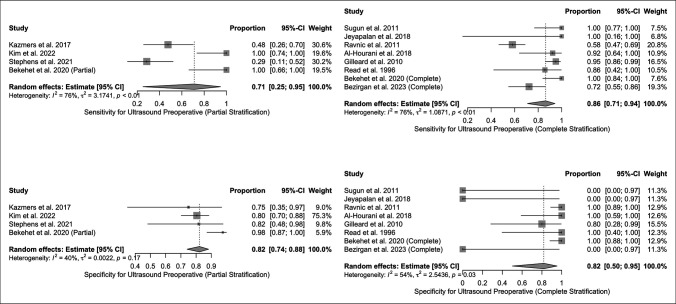
Sensitivities and specificities of US in the preoperative period stratified by partial and complete flexor tendon lacerations.

We also conducted statistical analyses to identify if any differences in accuracy existed when detecting partial versus complete lacerations (Figure 5). We were able to identify and stratify injuries by being either partial or complete in preoperative US; however, we were unable to make this stratification for MRI given that the reported studies either did not report enough outcomes for analysis or did not stratify sufficiently by laceration type. Heterogeneity was significant in pooled sensitivities for both partial laceration (I^2^ and τ^2^ of 76% and 3.17, respectively; *P* < 0.01) and complete laceration (I^2^ and τ^2^ of 76% and 1.09, respectively; *P* < 0.01) types. Although heterogeneity was also significant in pooled specificities for complete lacerations (I^2^ and τ^2^ of 54% and 2.54, respectively; *P* < 0.01), there was no significant heterogeneity in the partial laceration group (I^2^ and τ^2^ of 40% and 0.002, respectively; *P* = 0.17). When controlling for random effects, there were no statistically significant differences in sensitivity (*P* = 0.40) or specificity (*P* = 0.98) for partial lacerations in the preoperative period.

Diagnostic odds ratios (DORs) for US preoperative, US postoperative, MRI preoperative, and MRI postoperative, were 3.73 [2.26 to 5.20], 1.81 [0.73 to 2.88], 2.62 [1.13 to 4.11], and 4.06 [1.89 to 6.23], respectively (Supplemental Figure S1, http://links.lww.com/JG9/A462). There were no statistically significant differences between DORs in US and MRI preoperatively or postoperatively (*P* > 0.05). sROCs for both US and MRI in the preoperative and postoperative periods are outlined in Supplemental Figure S2, http://links.lww.com/JG9/A463. The area under the curve (AUC) for each of the imaging modalities were US preoperative: 0.92, US postoperative: 0.81, MRI preoperative: 0.83, and MRI postoperative: 0.91.

## Discussion

This systematic review and meta-analysis evaluates the true accuracies of US and MRI in preoperative and postoperative management of flexor tendon injuries by measurement of sensitivity, specificity, DORs, and sROCs with subsequent AUCs.

The diagnostic odds ratios of both imaging modalities in the preoperative and postoperative period suggest that either US or MRI are reasonable choices in the detection of flexor tendon injuries or the identification of postoperative complications. The AUCs in our generated sROCs also confirm that US and MRI can both be acceptable to use in both the preoperative and postoperative period. The small sample sizes in MRI preoperative, as well as US preoperative and postoperative, suggest that further primary research is required to confirm these findings.

The large heterogeneities in reported sensitivities for US and MRI preoperative and postoperatively, as well as specificities of US and MRI preoperatively, indicate the need for more standardized approaches to testing the true accuracies of these imaging modalities in management of flexor tendon injuries.

The similarities in sensitivity and specificity for US versus MRI in the pre and postoperative period suggests that US alone may be a sufficient imaging modality for making decisions regarding surgical intervention in the setting of flexor tendon injuries. This approach would prevent delayed surgical intervention in the many cases where MRI is not available or has a long wait time. Again, our large heterogeneities in reported results here indicate that further primary research would be beneficial in solidifying this finding.

The studies pooled for analysis indicated MRI to be markedly more specific than US for imaging of flexor tendon repairs in the postoperative period. These studies assessed accuracy in the identification of tendon adhesions, tendon rupture, and gap formation following tendon repair. In a clinical context, the increased specificity of MRI suggests a decreased rate of false positives in the postoperative assessment of tendon repair. Our findings therefore indicate that MRI may be a favorable choice of imaging modality in the postoperative period, as a decrease in false-positive detection of tendon adhesion, rerupture, and gap formation will prevent unnecessary clinic visits, patient anxiety, or reintervention in the form of repeat surgery.

One of the main strengths of this systematic review and meta-analysis is the large Cohen Kappa returned by article selection indicating substantial agreement between reviewers in both the screening and full-text review stages. This indicates that our research question was pointed and that screeners had a strong understanding of the objectives of the systematic review. Our paper is also strengthened by the robust statistical methods that have been verified by previous peer-reviewed literature. Finally, all the studies analyzed included a validation of the imaging findings to real-world surgical exploration and cadaveric investigation.

One limitation of our paper is the many comparisons conducted. Although we strictly followed peer-reviewed and established statistical methods, the many different calculations conducted may have increased the risk of false-positive findings. Clinical correlation and comparing our findings to existing literature is therefore paramount when interpreting our conclusions. The study is also limited by high heterogenicity found between the different imaging modalities in either the preoperative or postoperative period. It is important to also note that US accuracy is operator dependent, and we did not have data available to stratify by experience-level of those conducting the US. Finally, although this systematic review provides pooled estimates of sensitivity and specificity for both MRI and US, most included studies include a single imaging modality and as such do not offer head-to-head comparisons. Of the retrieved articles, only three directly compared US and MRI on patients in the same clinical or experimental conditions. This reflects a broader gap in the existing literature, given that most available studies evaluate single modalities and underscores the need for further research that provides direct head-to-head comparisons in the same patient cohorts.

## Conclusion

Although imaging modalities can be vital surgical decision-making tools for flexor tendon injuries, US and MRI are both largely underutilized given poor availability and concerns about accuracy. Large heterogeneities exist in sensitivity and specificity when comparing accuracy of US and MRI in identification of preoperative flexor tendon injury, underscoring the need for standardized comparisons in future literature. MRI is likely more specific than US in the postoperative period for identification of adhesion formation, rerupture, and gap formation. Postoperative US and preoperative MRI both have good diagnostic performance (AUC > 0.8), whereas preoperative US and postoperative MRI exhibit excellent diagnostic performance (AUC > 0.9).

## Supplementary Material

**Figure:**
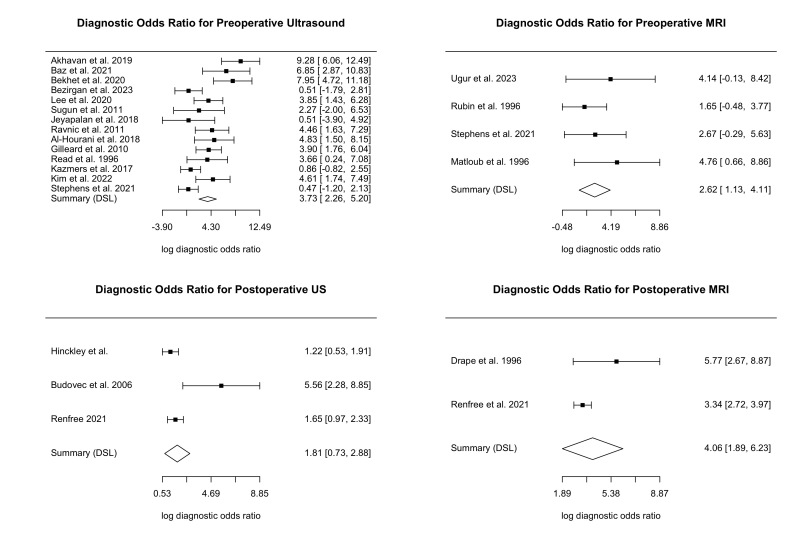


**Figure:**